# Group B streptococcus tricuspid endocarditis presenting with arthralgia in a postpartum woman: a case report

**DOI:** 10.1186/1752-1947-6-242

**Published:** 2012-08-14

**Authors:** Paul Vincent, Russell Davis, Debashis Roy

**Affiliations:** 1 Sandwell General Hospital, Lyndon West Bromwich, West Midlands, B71 4HJ, UK

## Abstract

**Introduction:**

Infective endocarditis presenting with arthralgia is rare. Group B streptococcus tricuspid endocarditis as a postpartum complication is even rarer. The present case is an example of both.

**Case presentation:**

We report the case of a 30-year-old Caucasian woman who presented with painful swelling of her wrists and ankles.

**Conclusion:**

Even when the clinical presentation of systemic inflammation is more suggestive of a primary rheumatological disorder, it is important to remember that bacterial infection can also present in this manner. Group B streptococcus tricuspid valve endocarditis is a rare, but recognized, postpartum complication.

## Introduction

Many diseases feature multisystem involvement. Diagnosing these diseases correctly is challenging enough. However, a particular conundrum occurs when the atypical presentation of one condition happens to fit the diagnostic criteria for a different condition. If the two conditions have completely different managements, it becomes a matter of life and death. Such a case is described here.

## Case presentation

A 30-year-old Caucasian woman presented to our Accident and Emergency department with immobility due to back pain. She reported a 10-day history of lumbar back pain, which started three days after giving birth. The pain was worse with movement and radiated down her legs. After seven days, the pain spread to involve her wrists, elbows, knees and ankles.

Our patient’s past medical history included only recent childbirth. Two weeks previously, she had given birth at 40 weeks to a healthy baby boy. Other than some light bleeding in the first trimester, there had been no antenatal symptoms or abnormalities on routine blood work or scans. She had no history of unexplained fever, sweats, vaginal discharge or pelvic tenderness. The delivery was unremarkable other than having required an episiotomy just before delivery because of a second-degree tear. One day after the delivery, our patient had complained of dysuria and was treated with trimethoprim for a urinary tract infection. The baby had not required hospitalization after delivery. He was fit and well at the time of our patient’s admission. There was no previous or family history of any autoimmune conditions, specifically primary arthropathies.

Initial observations revealed mild tachycardia only (heart rate, 107 beats per minute). No fever was detected. A clinical examination revealed warm, red, swollen wrists, forearms and shins and stiff elbows, ankles and knees with restricted ranges of motion. Heart sounds I and II were present with no additional sounds. Her breath sounds were normal and vesicular without any crepitations or bronchial breathing. An abdominal examination was unremarkable. A vaginal examination revealed a mildly tender cervix, but no vaginal discharge.

Results of initial investigations included a platelet count of 13×10^9^/L (normal range: 150×10^9^ to 450×10^9^/L), C-reactive protein of 312mg/L (normal range: <5mg/L), albumin of 22g/L (normal range: 35 to 50g/L), urea of 20mmol/L (normal range: 2.5 to 6.7mmol/L) and creatinine of 100μmol/L (normal range: 44 to 133μmol/L). A urine dipstick was positive for blood, protein and leukocytes and negative for nitrites. A urine culture from two weeks before had grown *Escherichia coli* that was sensitive to nitrofurantoin and trimethoprim. Results of other initial investigations are shown in Table 1.

**Table 1:** Results of initial investigations


Haemoglobin (11.5-15 grams/decilitre)	9.9	Albumin (35-50 grams/Litre)	22
White cell count (4-11 10^9^/Litre)	5.9	Bilirubin (<21 micromoles/Litre)	33
Platelets (150-450 10^9^/Litre )	13	Alanine Aminotransferase (<41 international units/Litre)	32
Mean Cell Volume (79-99 femtolitres)	85.7	Alkaline Phosphatase (20-130 international units/Litre)	254
International Normalised Ratio	1.27	C-Reactive Protein (<5 milligrams/Litre)	312
Urea (2.5-6.7 millimoles/L)	20	Erythrocyte Sedimentation Rate (1-12 millimetres/hour)	95
Creatinine (44-133 millimoles/Litre)	100		
Sodium (135-145 millimoles/Litre)	137	Electrocardiogram	Sinus tachycardia
Potassium (3.5-5 millimoles/Litre)	5.2	Chest X Ray	Normal

Routine investigations upon admission showed marked systemic inflammation, renal failure, thrombocytopenia and hypoalbuminemia.

In view of the unexplained inflammation with renal, joint and hematological involvement, a provisional diagnosis of systemic lupus erythematosus (SLE) was made. Differential diagnoses included occult sepsis (puerperal or urinary being considered most likely), HELLP syndrome (hemolysis, elevated liver enzymes and low platelet count), and a primary autoimmune nephropathy.

Our patient was started empirically on broad-spectrum intravenous antibiotics (gentamicin and amoxicillin with clavulanic acid) while confirmatory investigation results were pending.

On the second day of admission, our patient’s level of consciousness decreased to Glasgow Coma Scale (GCS) 10, with delirium and agitation. This was considered to possibly represent either neurological involvement in SLE or delirium due to sepsis. Antibiotic coverage was changed to intravenous ceftriaxone and acyclovir. Bacterial meningitis, viral encephalitis and thrombotic thrombocytopenic purpura were added to the differential diagnoses.

An urgent computed tomography scan of our patient’s head was performed, which showed no abnormalities. Our patient was transferred to our Critical Care department for observation of her consciousness level, which returned to GCS 15 over the course of several hours. A lumbar puncture was considered, but was not performed because of persisting profound thrombocytopenia.

By the end of day 3 after admission, a diagnosis had still not been made. There was no antibody evidence of SLE or any other autoimmune process. Blood films and bone marrow aspiration had shown no evidence of hemolysis or malignancy, two sets of blood cultures and a vaginal swab had been negative, and an abdominal ultrasound had failed to reveal a source of infection. Blood films showed toxic granulation of neutrophils, consistent with systemic sepsis. A complete list of investigations up to this point can be seen in Table 2.

**Table 2:** Further investigations available by the third day of admission

**Microbiology**		**Haematology**	
Mid Stream Urine	No organisms	Blood film	Toxic granulation of neutrophils. No schistocytes seen
High vaginal swab	No growth	Dilute Russell Viper venom test (<1.2)	1.99
cervical chlamydia screen	Negative	Fibrinogen (2-6 grams/Litre)	9.1
Syphilis antibodies	Negative	D-dimer (<0.5 microgram Fibrinogen Equivalent Units/ml)	18.22
Antistrepsolysin O titres	Negative	C3 (0.75-1.75 grams/Litre)	1.36
Parvovirus IgM	Negative	C4 (0.14-0.54 grams/Litre)	0.07
Mycoplasma antibodies	1:320 milli-International	Bone marrow aspirate	Reactive and fibrotic changes only. No malignant cells
(<1:320 mIU/ml)			
	Units per millilitre		
Blood cultures x 3	No growth	Ferritin (10-300 nanograms/millilitre)	738
Methicillin Resistant Staphylococcus Aureus nose and groin swabs	Negative	Direct Coombs test	Negative for anti IgG Weakly positive for anti C3b/d
Human Immunodeficiency Virus 1+2	Negative		
Herpes Simplex Virus Polymerase Chain Reaction 1+2	Negative		
**Immunology**		**Biochemistry**	
Anti Nuclear Antibody	Negative	Total protein (60-80 grams/Litre)	46
Anti Neutrophil Cytoplasmic Antibody	Negative	CK (<167 International Units/Litre)	316
Anti double-stranded deoxyribonucleic acid (DNA)	Negative	Urine albumin/Creatinine ratio (<2.26 milligrams/millimole)	4.87
Rheumatoid factor (<20 International Units/millilitre)	<11 International Units/millilitre	LDH (<225 International Units/Litre)	314
IgG	10.3		
(5.3-16.5 grams/Litre)			
IgA	2.26	**Radiology**	
(0.8-4 grams/Litre)			
IgM	3.08	Computed Tomography head	Normal
(0.5-2.5 grams/Litre)			
Urine Bence Jones protein	Negative	Ultrasound abdomen	Normal
Cardiolipin IgG	2.3	Computed Tomography abdomen	Distended uterus, in keeping with post-partum changes; otherwise normal.
(<10 anti-IgG phospholipid units/millilitre)			

Although a diagnosis was still lacking, results at this time pointed more toward severe sepsis than any other of our differentials.

Although still without a diagnosis, the bone marrow aspirate and blood film results and low reticulocyte count were more consistent with severe sepsis causing bone marrow suppression than an autoimmune hemolytic process. Along with the negative autoimmune tests, SLE was now considered less likely. The significance of the borderline positive mycoplasma serology was doubtful. Doxycycline was added to the antibiotic regimen, and plans were made to repeat the titers in one to two weeks.

**Figure 1:**
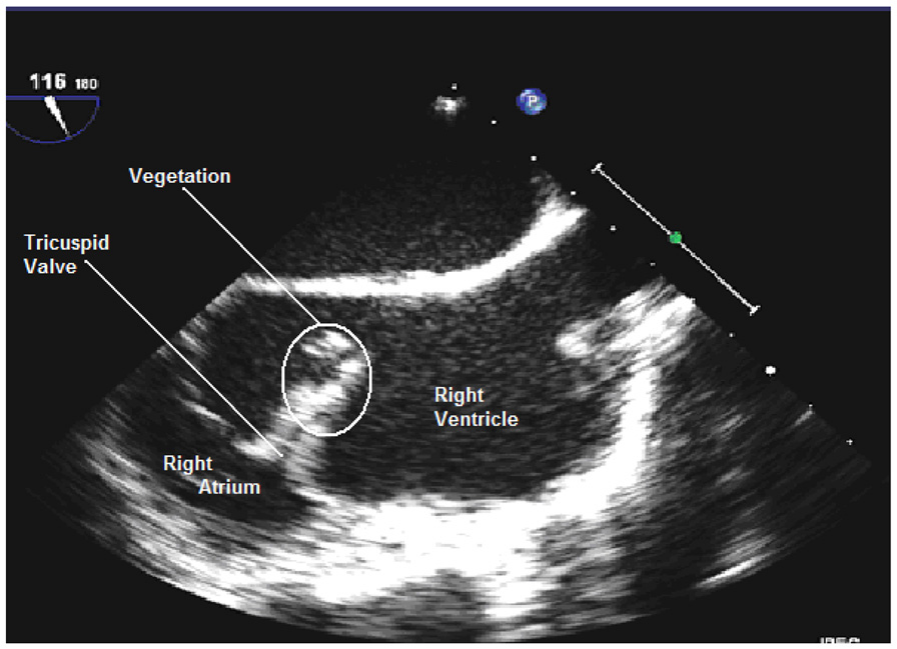
**Transesophageal echocardiogram image of tricuspid vegetation.** After initial imaging of the vegetation by a transthoracic echocardiogram, a transesophageal echocardiogram was performed to rule out an associated root abscess and in anticipation of a preoperative work-up.

On the evening of day 3 after admission, our patient unexpectedly developed sinus bradycardia at a rate of 35 beats per minute. A transthoracic echocardiogram revealed a large echogenic mass attached to the anterior leaflet of her tricuspid valve that was consistent with a vegetation or thrombus, moderate tricuspid regurgitation, a mildly dilated right heart with reduced right ventricular function, and mild left ventricular systolic impairment (Figure 1).

Our patient was re-examined by two cardiologists and a cardiothoracic surgeon for clinical evidence of endocarditis. No murmur was identified. There was some speculation as to whether or not there were two splinter hemorrhages on the left thumbnail, but this was not considered definitive.

A computed tomographic pulmonary angiogram was performed to look for septic emboli and showed widespread bilateral subsegmental emboli across both lungs. Her antibiotic coverage was changed again to daily gentamicin, ceftriaxone and vancomycin. A plan was made to treat with two weeks of intravenous antibiotics with the current regimen, then to repeat the echocardiogram.

After two weeks, a repeat echocardiogram showed no significant change, nor was there inflammatory resolution: her C-reactive protein level, after initially decreasing, remained consistent at >300mg/L (normal range: <5mg/L). Our patient’s arthralgia also failed to improve. Repeat mycoplasma serology failed to show rising titers, confirming suspicion that the borderline positive result was not significant. Table 3 shows her blood test results two weeks after admission.

**Table 3:** Blood results two weeks after admission


Haemoglobin (11.5-15 grams/decilitre)	7.7
Platelets (150-450 10^9^/Litre )	379
Mean Cell Volume (79-99 femtolitres)	89.2
White cell count (4-11 10^9^/Litre)	4.6
Albumin (35-50 g/Litre)	21
Bilirubin (<21 micromoles/Litre)	27
Alkaline Phosphatase (20-130 International Units/Litre)	217
Alanine Aminotransferase (<41 International Units/Litre)	22
Urea (2.5-6.7 millimoles/Litre)	10.2
Creatinine (44-133 micromoles/Litre)	78
Sodium (135-145 millimoles/Litre)	139
Potassium (3.5-5 millimoles/Litre)	4.8
C-Reactive Protein (<5 milligrams/Litre)	349
Blood cultures (5 sets sent in total)	No growth
Mycoplasma serology (<1:320 milli-International	Day 2 of admission 1:320
Units per millilitre)	Day 9 of admission 1:320
	Day 15 of admission 1:320

Although the patient’s profound thrombocytopenia had resolved, antibiotic therapy had failed to affect the increased C-reactive protein levels or hypoalbuminemia.

For definitive treatment, our patient was scheduled for tricuspid valve removal and replacement. Sixteen days after her initial admission, she was transferred to a specialist cardiothoracic center. Operative findings included pericardial effusion, severe right atrial dilatation, a volume-loaded right ventricle, and a large vegetation on the anterior and posterior leaflets of her tricuspid valve. The mural leaflet was spared. Her tricuspid valve was removed, and a 27mm biological valve was inserted.

A postoperative echocardiogram showed good valve function with a small amount of paravalvular leakage. Pericardial fluid and vegetation microscopy, sensitivity and culture failed to identify a pathogen. A sample of the vegetation was sent to a reference laboratory. The pathogen was eventually identified by 16S ribosomal deoxyribonucleic acid (DNA) sequence analysis as Streptococcus Lancefield Group B (GBS; *Streptococcus agalactiae*).

The source remained unclear; there was no clinical evidence of chorioamnionitis or infection of the episiotomy incision. Asymptomatic infection at either of these sites could have caused hematogenous spread. Another possibility is transient bacteremia from superficial colonization with Group B Streptococcus, without a primary local infection. Antenatal screening for Group B Streptococcus colonization is not routine in the UK and was not performed in our patient.

Our patient was continued on the antibiotic regimen of gentamicin, vancomycin and ceftriaxone for an additional 22 days. After this period, all inflammatory markers had normalized. Our patient’s original polyarthritis finally resolved. At the three-month follow-up, all abnormal blood parameters had returned to normal levels (Table 4). Our patient was pain-free with a normal exercise tolerance.

**Table 4:** Blood results three months post-valve replacement


Haemoglobin (11.5-15 grams/decilitre)	15.8
White cell count (4-11 10^9^/Litre)	6.7
Platelets (150-450 10^9^/Litre )	211
Mean Cell Volume (79-99 femtolitres)	87.4
Albumin (35-50 g/Litre)	52
Bilirubin (<21 micromoles/Litre)	6
Alanine Aminotransferas (<41 International Units/Litre)	29
Alkaline Phosphatase (20-130 International Units/Litre)	102
Urea (2.5-6.7 millimoles/Litre)	5
Creatinine (44-133 micromoles/Litre)	66
Sodium (135-145 millimoles/Litre)	143
Potassium (3.5-5 millimoles/Litre)	3.9
C-Reactive Protein (<5 milligrams/Litre)	<5
Dilute Russell Viper venom test (<1.2)	1.14

Results at three months showed complete resolution of abnormal results.

## Discussion

This case is a reminder of the heterogeneity of conditions that can present with musculoskeletal symptoms. Practically any infectious process may cause a reactive polyarthritis, although this is more common with some organisms (for example, *Chlamydia*) than others.

It is important to remember that it is not uncommon for malignancies to present with musculoskeletal symptoms. This may occur by various mechanisms, including bone pain from metastases or lytic lesions, paraneoplastic polyarthritis (mostly seen in breast, lung and renal cell carcinoma), or polymyositis and/or dermatomyositis.

This case also highlights the need for a systematic approach to diagnose inflammation of an unknown origin. A useful approach is to follow that of pyrexia of unknown origin [1], the main diagnosis categories of which are infection, autoimmune disease and malignancy.

Infective endocarditis is a relatively uncommon condition that, if undiagnosed, leads to serious morbidity and mortality [2]. There are many documented instances of infective endocarditis presenting in an atypical fashion [3,4]. This case presented a particularly difficult diagnostic challenge. At no point in this patient’s admission did she display even a low-grade fever (the most sensitive clinical symptom or sign [5]), an increased neutrophil count, a murmur or clinical signs of heart failure.

Furthermore, our patient met the diagnostic criteria for SLE, displaying four of the eleven diagnostic features: non-erosive arthritis, hematological manifestation (thrombocytopenia), renal involvement (proteinuria) and neurological involvement (acute confusional state).

GBS endocarditis is associated with pregnancy and older patients with comorbidities. Our patient showed typical complications of mild systolic dysfunction of both ventricles and requirement of valve replacement.

GBS is a common pathogen in puerperal sepsis. A correlation between pregnancy and GBS tricuspid endocarditis is therefore conspicuous by its absence: a literature review revealed only seven cases of pregnancy-associated GBS tricuspid endocarditis [6].

Because GBS screening is not routinely performed in the UK, our patient was not offered antenatal screening. Because of the rarity of GBS endocarditis, it is doubtful whether a positive result would have given an indication of the diagnosis. A debate of the benefits of antenatal GBS screening is beyond the scope of this case report. GBS endocarditis is certainly not a common enough postpartum complication to be used as an argument in favor of screening.

## Conclusion

We report an atypical presentation of infective endocarditis presenting with features of an inflammatory polyarthropathy. This case highlights the importance of considering an empirical investigation for a bacterial infection in any patient with unexplained biochemical evidence of inflammation, even in the presence of clinical features suggesting a primary rheumatological disorder.

## Consent

Written informed consent was obtained from the patient for publication of this case report and accompanying images. A copy of the written consent is available for review by the Editor-in-Chief of this journal.

## Competing interests

The authors declare that they have no competing interests.

## Authors’ contributions

PV collated clinical details and investigation results of the case and was the primary author of the manuscript. RD performed imaging of the tricuspid valve, chose a still image for submission, and was a major contributor to the literature review. DR was a major contributor in establishing the narrative of the case presentation. All authors read and approved the final manuscript.
